# Stratification of Homologous Recombination Deficiency-Negative High-Grade Ovarian Cancer by the Type of Peritoneal Spread into Two Groups with Distinct Survival Outcomes

**DOI:** 10.3390/cancers16112129

**Published:** 2024-06-03

**Authors:** Simon Schnaiter, Esther Schamschula, Juliane Laschtowiczka, Heidelinde Fiegl, Johannes Zschocke, Alain Zeimet, Katharina Wimmer, Daniel Reimer

**Affiliations:** 1 Institute of Human Genetics, Medical University Innsbruck, 6020 Innsbruck, Austria; esther.schamschula@i-med.ac.at (E.S.); juliane.laschtowiczka@i-med.ac.at (J.L.); johannes.zschocke@i-med.ac.at (J.Z.); katharina.wimmer@i-med.ac.at (K.W.); 2 Department of Obstetrics and Gynecology, Medical University Innsbruck, 6020 Innsbruck, Austria; heidelinde.fiegl@i-med.ac.at (H.F.); alain.zeimet@i-med.ac.at (A.Z.); daniel.reimer@i-med.ac.at (D.R.)

**Keywords:** personalized medicine, genomic instability, miliary disease, epithelial ovarian carcinoma, PARP inhibitor, staging

## Abstract

**Simple Summary:**

Homologous recombination deficiency plays an important role in high-grade ovarian cancer development and is used as an important marker to predict cancer survival based on the patient’s response to treatment. The present study used a new score that we developed to assess homologous recombination deficiency, called the predictive-value integrated genomic instability score. We then looked at two different types of cancer spread (miliary versus non-miliary) and saw that tumors with miliary type tumor spread were negative for homologous recombination deficiency and that patients with miliary type tumor spread had a much poorer likelihood of survival than patients with non-miliary spreading tumors, independent of homologous recombination deficiency. This suggests that the type of tumor spread is the dominant predictor of the response to cancer therapy and homologous recombination deficiency is an indirect marker for the type of tumor spread.

**Abstract:**

Background: Homologous recombination deficiency (HRD) has evolved into a major diagnostic marker in high-grade ovarian cancer (HGOC), predicting the response to poly (adenosine diphosphate-ribose) polymerase inhibitors (PARPi) and also platinum-based therapy. In addition to HRD, the type of peritoneal tumor spread influences the treatment response and patient survival; miliary type tumor spread has a poorer predicted outcome than non-miliary type tumor spread. Methods: Known methods for HRD assessment were adapted for our technical requirements and the predictive-value integrated genomic instability score (PIGIS) for HRD assessment evolved as an outcome. PIGIS was validated in HGOC samples from 122 patients. We used PIGIS to analyze whether the type of tumor spread correlated with HRD status and whether this had an impact on survival. Results: We demonstrated that PIGIS can discriminate HRD-positive from HRD-negative samples. Tumors with a miliary tumor spread are HRD-negative and have a very bad prognosis with a progression-free survival (PFS) of 15.6 months and an overall survival (OS) of 3.9 years. However, HRD-negative non-miliary spreading tumors in our cohort had a much better prognosis (PFS 35.4 months, OS 8.9 years); similar to HRD-positive tumors (PFS 34.7 months, OS 8.9 years). Conclusions: Our results indicate that in a predominantly PARPi naïve cohort, the type of tumor spread and concomitant cytoreduction efficiency is a better predictor of survival than HRD and that HRD may be an accidental surrogate marker for tumor spread and concomitant cytoreduction efficiency. It remains to be determined whether this also applies for sensitivity to PARPi.

## 1. Introduction

Ovarian cancer is the eighth most common cancer in women [1], in whom it accounts for 2.2% of all cancer deaths [2]. Epithelial ovarian cancer (EOC) is the most common cause of death in gynecologic cancers [3]. The 5-year survival rate of ovarian cancer is 50.8% [2,4]. Unfortunately, most EOCs are diagnosed at an already advanced stage (FIGO stage III-IV), and more than 85% are high-grade ovarian cancer (HGOC).

Two distinct modes of peritoneal spread of HGOC, miliary and non-miliary with bulky implants [5,6], can be distinguished during surgery and are highly predictive of the clinical outcome [5,7,8,9]. Miliary carcinomatosis, for example, on the small bowel, is associated with poor surgical outcomes [7,10], and it has been hypothesized that patients with miliary disease are less likely to respond to platinum therapy [7].

Approximately 50% of HGOC cases show genomic instability (GI) due to homologous recombination deficiency (HRD) [11]. HRD is caused by genetic and epigenetic alterations in the genes that encode key players of the homologous recombination repair (HRR) pathway. Among these, germline and/or somatic inactivating mutations in the *BRCA1* and *BRCA2* genes (13–15% of all HGOC) [11,12,13,14,15] are the most frequent. Other HRR genes affected include *RAD51C*, *RAD51D*, *PALB2*, and *ATM* [16,17], or different undetermined genomic alterations [18].

HRR is required for the accurate repair of DNA double strand breaks. HRR-deficient cells utilize alternative more error-prone DNA double strand repair mechanisms such as non-homologous end joining or mitotic homologous recombination [15]. Consequently, HRR-deficient cells display GI with the accumulation of copy number alterations (CNAs) and/or an increased number of regions with a loss of heterozygosity (LOH), resulting in characteristic genomic scars [18,19].

HRD plays an important role in HGOC oncogenesis, progression, and metastasis and thus represents a fundamental vulnerability that can be exploited in therapy [16,20]. Tumors exhibiting HRD are highly sensitive to DNA damaging agents such as platinum-based antitumor drugs and to selective inhibitors of poly (adenosine diphosphate -ribose) polymerase (PARPi) which induce synthetic lethality. Both favorably affect overall survival (OS) and progression-free survival (PFS) in HGOC [21,22]. HRD testing for prediction of the therapy response can focus on pathogenic variants in genes of the HRR pathway and/or the presence of characteristic genomic scar patterns [16]. Such scar patterns independently predict the response to the drugs mentioned above and to prognosis in ovarian cancer patients [16,23,24,25].

Different scores/algorithms are available to quantify the characteristic genomic scar patterns resulting from HRD. Amongst others, the loss of heterozygosity (LOH) score according to Abkevich [26] and the Telomeric Allelic Imbalance (TAI) score [27] are well-established markers for the determination of HRD.

To our knowledge, a possible link or correlation of peritoneal type of tumor spread and HRD status has not been investigated thus far. With the aim of closing this gap, we assessed if such a link or correlation between the peritoneal type of tumor spread and HRD status existed and whether this had an influence on the PFS and OS in a cohort of patients with HGOC. For the HRD assessment, we established a novel score based on an equally weighted combination of the well-established LOH score [26] and a modified TAI score [27].

## 2. Methods

### 2.1. Patients

The study was performed on samples collected by the Department of Obstetrics and Gynecology, at the Medical University Innsbruck, Austria, between 28 August 1990 and 1 September 2021.

Patient samples for the cohort were very stringently selected according to the criteria in Table 1.

One hundred and twenty-two samples from patients with HGOC were included in the study. The total number of HGOC patients treated in the study period (1990–2021) was 699. In total, 4 patients included in the study were first diagnosed from 1990–1999, 28 patients from 2000–2009, 68 patients from 2010–2019, and 22 patients from 2020–2021.

The ECOG (Eastern Co-operative Oncology Group) performance status for most patients (70.5%) was “0” (*n* = 86), indicating that the majority of patients were “fully active, able to carry on all pre-disease performance without restriction”. The predominant histology sub-type was high-grade serous (HGS) (89.3%; *n* = 109); with approximately one third (34.4%; *n* = 42) graded “Grade 2” (moderate or intermediate grade), and two thirds (65.6%, *n* = 80) Grade 3 (high-grade or poorly differentiated). The HGOC samples included were staged according to the International Federation of Gynecology and Obstetrics (FIGO) classification from 2017. Most patients were classified as Stage III (72%; *n* = 88) or Stage IV (a/b) (19.7%; *n* = 24). A complete summary of the patients’ clinicopathologic parameters are shown in Table 2.

The mean age at diagnosis was 61.32 (38.65–86.07) years. The majority, 77.9% of patients (*n* = 95), received primary debulking surgery (PDS), whereas 22.1% of patients (*n* = 27) received neoadjuvant chemotherapy followed by interval debulking surgery (IDS). Adjuvant treatment consisted of six cycles of paclitaxel (175 mg/m^2^) and carboplatin (AUC5). Neoadjuvant treatment consisted of three cycles of paclitaxel (175 mg/m^2^) and carboplatin (AUC5) before and after interval debulking. The overall complete macroscopic tumor resection rate was 76.2% (*n* = 93). Adjuvant platinum-based therapy was performed in all patients within the collective, whereby 81.0% (*n* = 99) of individuals received a paclitaxel–carboplatin combination. In addition, 54.1% (*n* = 66) of patients were subjected to maintenance therapy, mostly bevacizumab 15 mg/kg body weight for 15 months or until progression. A summary of the patient treatment characteristics is shown in Table 3, including the patients’ recurrence rate, mutational status, and peritoneal spread.

During a median follow-up of 2.92 (1.45–5.42) years, recurrence was observed in 68.9% of patients (*n* = 84), and 40.2% of patients died (*n* = 49), resulting in a mean PFS of 32 (0.4–187.0) months, and a mean OS of 3.88 (0.03–15.59) years.

The mutation status of cancer specimens was assessed in previous research projects at the Department of Obstetrics and Gynecology, Medical University Innsbruck, Austria, or during tumor profiling at the Institute of Human Genetics, Innsbruck Medical University, Austria. In our cohort, 27.9% (*n* = 34) of the tumors harbored pathogenic variants (PVs) in *BRCA1*, 8.2% (*n* = 10) in *BRCA2*, and 4.1% (*n* = 4) in *RAD51C*. The cohort was initially designed to setup HRD testing and therefore enriched in samples containing *BRCA1*/*2* mutations (36%).

### 2.2. Ethical Approval

Clinical, pathological, and follow-up data were stored in a database according to the hospital’s data privacy regulations. Patients gave written informed consent for the collection of their data for research purposes. The study was conducted according to the guidelines of the Declaration of Helsinki, and approved by the Ethics Committee of the Innsbruck Medical University (Reference Numbers: AN2015-0038 346/4.17, 16 March 2015; 1054/2019, 27 June 2019).

### 2.3. Type of Peritoneal Spread

The type of peritoneal spread was determined independently by two surgeons during primary surgery according to the criteria as described by Auer et al. [6]. Briefly, peritoneal spread was designated as miliary if more than 20 (frequently uncountable) implants with a diameter smaller than two centimeters in the majority of the deposits were observed. Cases with easily numerable large noduled implants with an exophytical growing pattern, mostly larger than two centimeters in diameter, were allocated to the group of non-miliary spreading cancers. In total, 74.6% of patients were classified as non-miliary (*n* = 91) and 25.4% were classified as miliary (*n* = 31).

### 2.4. SNP-Array and Data Analysis

The DNA from the 122 tumor samples was extracted from fresh frozen tissue (>20% tumor cell content), or from formalin-fixed, paraffin-embedded (FFPE) tissue (>50% tumor cell content). The DNA was analyzed using the Global Screening Array (Illumina) according to the manufacturer’s protocol. To determine the HRD status (see “Scoring”), data were analyzed with Illumina GenomeStudio 2.0 and NxClinical (Bionano, San Diego, CA, USA, SNP-FASST2-Segmentation Algorithms).

### 2.5. Scoring (Integrated HRD Score)

To quantify HRD, a LOH score and an Aneuploidy Normalized Telomeric Imbalance (ANTI) score were used based on the LOH score published by Abkevich et al. [26], and using a modification of the TAI score of Birkbak et al. [27].

In brief, the LOH score was calculated as the percent of genomic regions spanning less than 90% of a chromosome arm, with a total allele copy number higher than zero and a minor allele copy number of zero.

The ANTI score was calculated based on the number of chromosomal regions meeting all of the following criteria: (1) starting at the end of the telomere, but not reaching the centromere (breakpoint within the chromosome arm); (2) major copy proportion (MCP (copy number (CN) of the major allele/(CN major allele + CN minor allele)) > 0.7; and, (3) minimal fragment size of 12 Megabases.

**Figure 1:**
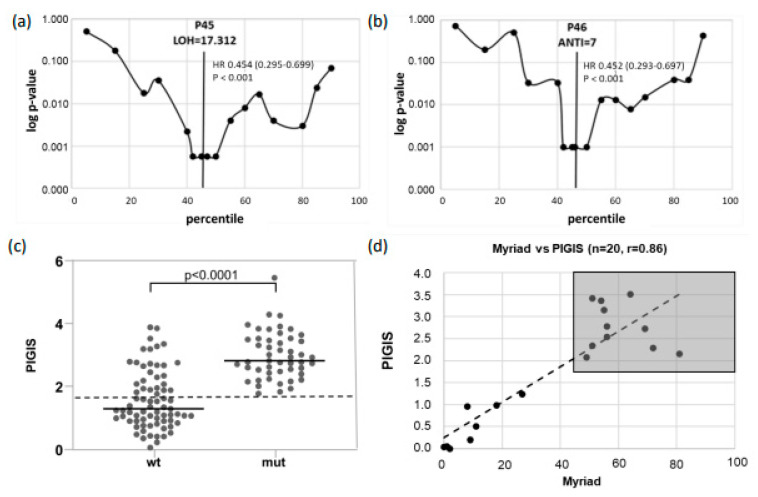
Setup of the predictive-value integrated genomic instability score (PIGIS): For determination of an optimal predictive cut-off point for (**a**) the loss of heterzygosity (LOH) score, and (**b**) the aneuploidy normalized telomeric imbalance (ANTI) score, the patients were separated into two groups for each score with cut-off points corresponding to every 5th percentile of the value of each score (*x*-axis). Kaplan–Meier estimations for PFS were calculated for the two groups at every 5th percentile (in the ranges p5–p40 and p50–p90) or every percentile (in the range of p41–p49). Logarithmic *p*-values (Log-rank test) for calculated groups are displayed on the *y*-axis. A vertical line indicates the optimal cut-off point for each score. The optimal cut-off point was at Percentile 45 (P45) for the LOH score (score: 17.312) (**a**) and at Percentile 46 (P46) for the ANTI score (score: 7) (**b**). Panel (**c**) shows the PIGIS scores for wild type (wt) and mutant (mut) samples. The diagnostic cut-off is indicated as a dashed black line. Correlation of PIGIS and Myriad MyChoiceDX (**d**). The gray rectangle indicates samples classified HRD-positive by both scores.

The optimal predictive cut-off values of both scores (LOH and ANTI) were determined as follows: For both scores, the patients were separated into two groups, LOH-high or LOH-low, and ANTI-high or ANTI-low, using 20 different cut-off points corresponding to every 5th percentile of the maximum score value. Kaplan–Meier estimations and hazard ratios (HRs) for PFS were calculated for each of these two groups. In the range with the lowest *p*-values (percentiles 40–50), Kaplan-Meier estimations and HRs for PFS were calculated for the two groups separately at each percentile. The score values of the percentile with the lowest *p*-values (Log-rank test) were selected as the optimal cut-off points (Figure 1a,b).

### 2.6. Statistical Analysis

Statistical analysis was performed using the Statistical Product and Service Solutions (SPSS) (Version 28.0.0, IBM, Armonk, NY, USA) program or with Prism (Version 10.1.1, Graphpad Software, Boston, MA, USA). A *p*-value of ≤0.05 was considered statistically significant.

## 3. Results

### 3.1. Setup of the Integrated HRD Score

The optimal cut-off point for the LOH score with the highest predictive significance of PFS was the 45th percentile (*p* < 0.001), corresponding to an LOH score of 17.31% (Figure 1a). For the ANTI score, the optimal threshold value for PFS prediction was the 46th percentile (*p* < 0.001) corresponding to an ANTI score of 7.0 (Figure 1b).

To integrate the LOH and the ANTI score in one single HRD score termed the “predictive-value integrated genomic instability score” (PIGIS), the two relative scores (individual score of the patient divided by the optimal predictive cut-off values) were added.

$$\mathrm{PIGIS}=\frac{LOH-score}{LOH-cutoff}+\frac{ANTI-score}{ANTI-cutoff}$$

### 3.2. Determination of a Diagnostic Threshold of PIGIS

Samples with *BRCA1/2*- or *RAD51C*-mutations (mut) had a significantly higher median PIGIS (*p* < 0.0001, Wilcoxon signed rank test) than the other samples in the cohort (wt) (Figure 1c). In previous studies [24], the cut-off for HRD positivity was set at the HRD value corresponding to the 5th percentile of samples harboring *BRCA1/2*-mutations. The 5th percentile was selected to compensate for outliers, e.g., samples with *BRCA1/2*-mutations without chromosomal instability. However, since our cohort contained no outliers, the PIGIS threshold for HRD positivity was defined as the lowest PIGIS value in a sample harboring a PV in *BRCA1/2* or *RAD51C* (Figure 1c). Using this PIGIS cut-off value (1.77), 25 samples without *BRCA1/2*- or *RAD51C*-mutations were HRD-positive (Figure 1c). Testing of a proportion of the cohort (*n* = 20) with Myriad MyChoice DX (Myriad Genetics, Salt Lake City, UT, USA) showed a good correlation, with a Pearson score of 0.86; the classification of samples into HRD-positive and HRD-negative was the same for all samples (Figure 1d).

### 3.3. Patients with PIGIS (HRD)-Positive Tumors Have Better Prognosis

**Figure 2:**
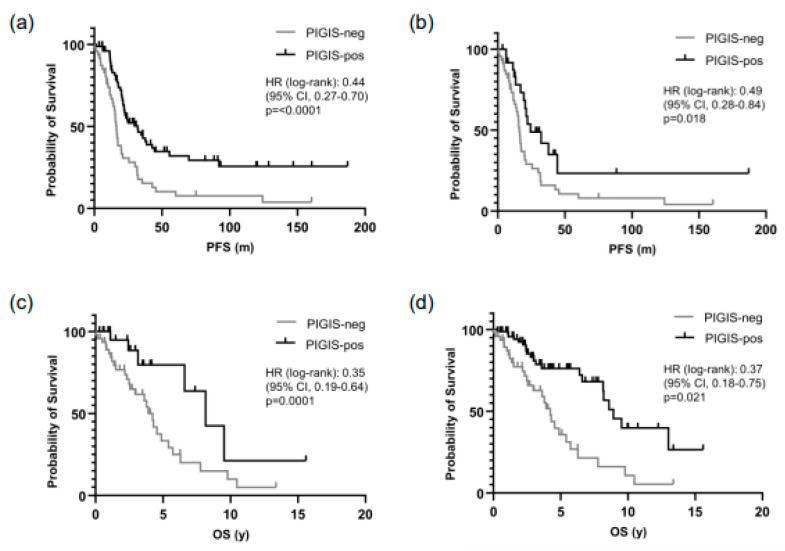
The survival prediction by the predictive value integrated genomic instability score (PIGIS) in (**a**) the progression-free survival (PFS) of patients with PIGIS-positive tumors vs. PIGIS-negative tumors; (**b**) the PFS of patients with PIGIS-positive tumors with no *BRCA1/2* or *RAD51C* mutations vs. PIGIS-negative tumors; (**c**) the overall survival (OS) of patients with PIGIS-positive tumors vs. PIGIS-negative tumors, and (**d**) the OS of patients with PIGIS-positive tumors with no *BRCA1/2* or *RAD51C* mutations vs. PIGIS-negative tumors.

Patients with HRD-positive tumors (PIGIS value ≥ 1.77), had significantly better survival than patients with HRD-negative tumors (PIGIS value < 1.77) with a median PFS of 32.0 vs. 16.4 months (HR 0.44, *p* < 0.0001) (Figure 2a). Excluding patients having tumors with *BRCA1*/*2*- or *RAD51C*-mutations from the analysis, patients with HRD-positive tumors still had a significantly better survival than patients with HRD-negative tumors, with a median PFS of 24.4 vs. 16.4 months (HR 0.49, *p* = 0.018) (Figure 2b).

Similarly, OS was better in patients with PIGIS HRD-positive tumors, with an OS of 8.9 vs. 4.2 years (HR 0.35, *p* = 0.0001; Figure 2c). Also, excluding the patients with *BRCA1*/*2* or *RAD51C* PV-positive tumors in this analysis, the OS in the subgroup of HRD-positive tumors was significantly better than in patients with HRD-negative tumors (8.2 years vs. 4.2 years, HR 0.37, *p* = 0.02; Figure 2d).

### 3.4. Tumors with Miliary Spread Are Exclusively PIGIS-Negative and Have a Poorer Prognosis

**Figure 3:**
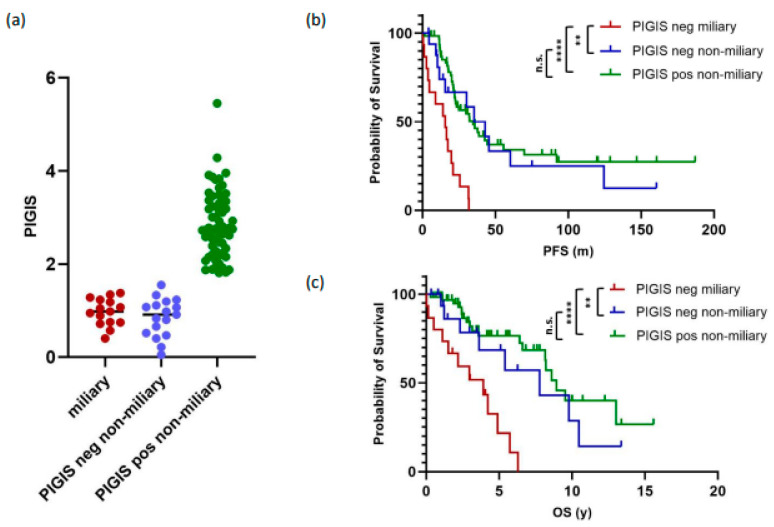
Analysis of miliary vs. non-miliary tumors showing (**a**) the distribution of individual predictive value integrated genomic instability score (PIGIS) values for miliary, non-miliary PIGIS-negative, and non-miliary PIGIS-positive tumors, (**b**) the progression-free survival (PFS) of patients with miliary, non-miliary PIGIS-negative, and non-miliary PIGIS-positive tumors, and (**c**) the overall survival (OS) of patients with miliary, non-miliary PIGIS-negative, and non-miliary PIGIS-positive tumors (explanation of symbols: n.s. = not significant; ** = *p*-value 0.001–0.009, **** = *p*-value ≤ 0.0001).

Separating the cohort into tumors without (*n* = 91) and with (*n* = 31) miliary spread revealed that tumors with miliary spread were exclusively HRD-negative (PIGIS value < 1.77; Figure 3a). This observation led us to compare whether patients with HRD-negative non-miliary tumors had a different clinical outcome to patients with HRD-negative miliary tumors. For this analysis, we only analyzed samples from patients that underwent primary debulking surgery, to avoid a bias in the classification of the peritoneal carcinogenesis due to potential differences in the response to neoadjuvant treatment. The analysis of PFS and OS showed that patients with HRD-negative non-miliary tumors (*n* = 17) had a similar clinical outcome to patients with HRD-positive tumors with a non-miliary spread (*n* = 62, median PFS 35.4 vs. 34.7 months, *p* = 0.6; median OS 7.8 vs. 8.9 years, *p* = 0.3; Figure 3b,c).

Patients with miliary spreading tumors (*n* = 15) had a significantly poorer prognosis than patients with HRD-positive tumors (median PFS 15.6 vs. 34.7 months, *p* ≤ 0.0001; median OS 3.9 vs. 8.9 years, *p* ≤ 0.0001), and HRD-negative non-miliary spreading tumors (median PFS 15.6 vs. 35.42 months, *p* = 0.001; median OS 3.9 vs. 8.9 years, *p* = 0.009; Figure 3b,c).

### 3.5. Residual Disease and Survival Correlates with Peritoneal Spread, but Not with HRD

**Figure 4:**
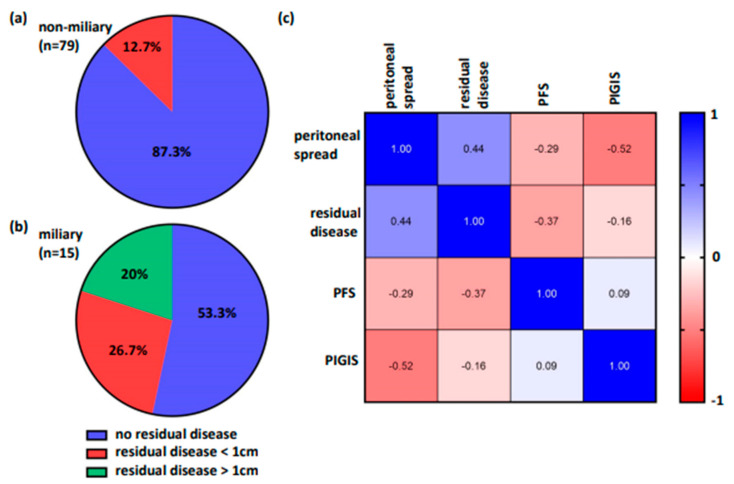
(**a,b**) Cytoreduction surgery is more successful in non-miliary tumors than in miliary tumors. (**c**) Peritoneal spread correlates significantly with residual disease, progression-free survival (PFS), and predictive value integrated genomic instability score (PIGIS). PIGIS significantly correlates with peritoneal spread, but not with residual disease and PFS.

In our cohort of patients with non-miliary tumor spread that underwent primary debulking surgery (*n* = 79), no residual disease was determined in 69 patients (87.3%), while 10 patients (12.7%), which were all HRD-positive, had residual tumors smaller than 1 cm (Figure 4a). In patients with miliary tumor spread that underwent primary debulking surgery (*n* = 15), no residual disease was determined in eight patients (53.3%), while four patients (26.7%) had residual tumors smaller than 1 cm, and three patients (20%) had residual tumors larger than 1 cm (Figure 4b).

Finally, we wanted to know if HRD, tumor spread, residual disease, and survival correlated. For this analysis, we limited our cohort to patients who had primary debulking surgery and suffered recurrence (*n* = 63). The type of peritoneal tumor spread correlated significantly with HRD (r = −0.52, *p* ≤ 0.0001), residual disease (r = 0.44; *p* = 0.0003), and PFS (r = −0.29; *p* = 0.019). Also, the residual disease and PFS correlated significantly (r = −0.37; *p* = 0.003). However, HRD only correlated with the type of peritoneal tumor spread (r = −0.52, *p* ≤ 0.0001), but not with residual disease (r = −0.16; *p* = 0.22) or PFS (r = −0.09; *p* = 0.5; Figure 4c).

## 4. Discussion

Using a newly developed and validated HRD score, denoted PIGIS, we show that all miliary spreading tumors in our cohort were HRD-negative and patients with such tumors had a significantly worse PFS and OS than patients with HRD-negative non-miliary spreading tumors. Patients with HRD-negative non-miliary spreading tumors in our cohort had a similar PFS and OS as HRD-positive tumors.

The identification of the HRD status of HGOC patients as being a reliable predictor of a high response to neoadjuvant platinum-based therapy and PARPi has led to the large-scale development of HRD testing. This was implemented either on a commercial basis (e.g., Myriad MyChoice, Foundation Medicine), as integrated kits for in-house testing (e.g., Illumina TruSight Oncology 500 DNA Kit, SOPHiA DDM™ Homologous Recombination Deficiency (HRD) Solution) or as local multi-factorial analysis for research and diagnostic purposes (e.g., the Leuven HRD test [28] or the Scarface Score [23]). The tests differ in cost, accessibility, and time of return; nonetheless, they are an integral part of tumor profiling for gynecological tumors. To improve accessibility, cost efficiency, and the time of return for our own in-house use, we established the novel PIGIS score, an equally weighted combination of the well-established loss of heterozygosity (LOH) score [26] and a modification of the TAI score [27] for HRD assessment. The goal of the integration of the two scores into one single parameter was to balance the potential weaknesses and strengths of both individual scores.

Samples harboring *BRCA1/2* mutations and *RAD51C* mutations had a significantly higher PIGIS than samples with no known PV in one of these genes, even though in the later cohort several samples with high scores were identified. These high-score samples without known *BRCA1/2* mutations or *RAD51C* mutations were expected and can most probably be explained by mutations in HRR genes other than *BRCA1/2* or *RAD51C* or the epigenetic silencing of HRR genes. Based on these data, we concluded that PIGIS is a valid score for the determination of HRD in HGOC samples.

Patients with HRD-positive tumors are known to have a better prognosis in comparison to patients with HRD-negative tumors [21,25]. Indeed, survival analysis in our cohort confirmed a better survival for patients with HRD-positive HGOC, underlining the validity of our testing approach.

The miliary spread pattern is a known negative predictor for OS in HGOC patients [5,6,7,10], which was confirmed in our cohort. Interestingly, we could also show that miliary spreading tumors were consistently HRD-negative. We hypothesized, that if a miliary tumor spread pattern is a negative predictor for survival, and all of these cancers are HRD-negative, the miliary pattern of intraperitoneal dissemination might be responsible for the generally worse survival (PFS and OS) of the HRD-negative tumor group. To test this hypothesis, we formed a miliary and non-miliary cohort out of the HRD-negative group and performed survival analysis. We found that patients with HRD-negative non-miliary tumors had a similar PFS and OS to patients with HRD-positive tumors, whilst patients with miliary spreading tumors, which were all HRD-negative, exhibited a much worse prognosis.

Previous studies suggest that patients with miliary tumor spread have a worse response to initial treatment, in surgery as well as with platinum-based chemotherapy [7,10]. Molecular characterization of miliary vs. non-miliary tumors, amongst other methods by transcriptome analysis including a pathway analysis of differentially expressed genes, supports the hypothesis that more mesenchymal-like characteristics in miliary spreading tumor cells are responsible for potential platinum resistance [5]. However, studies explaining the exact mechanism of resistance or whether the worse prognosis of patients with miliary tumor spread is dependent on platinum resistance or impaired surgical efficiency are yet lacking.

In our cohort, we showed that miliary tumor spread correlated with a worse efficiency in cytoreductive surgery, which favors the hypothesis that the impact on survival based on the type of tumor spread predominantly originates from impaired surgical success rather than impaired sensitivity to platinum-based therapy. The group of HRD-negative tumors contains the miliary spreading tumors, and consequently HRD correlates with the type of tumor spread. However, HRD does not correlate with residual disease and survival; implying that the predictive value of HRD originates from the miliary tumors contained in the group of HRD-negative tumors and is not directly related to HRD. Consequently, HRD is rather a surrogate marker for tumor spread, which in turn is a strong predictor for cytoreduction efficiency and concomitantly for survival.

As it was shown that the response to initial treatment, also called platinum sensitivity, is an intrinsic predictor of PARPi response [29], we speculate that the type of tumor spread could also be a valuable parameter, in combination with HRD or even alone, to predict the responsiveness to PARPi in HGOC. To elucidate this hypothesis, further studies in a larger cohort of patients treated with PARPi are needed.

Limitations exist within the study given that the outcomes have not been validated in an independent dataset. The cohort consists of only a small number of patients/samples, and the allocation to the miliary and non-miliary type with larger bulky implants remains a subjective assessment made by surgeons. This bears the risk of an intra- and inter-surgeon bias, which could lead to discrepancies in the interpretation of the type of tumor spreading, even if, as in our cohort, the assessment was made independently by two surgeons.

Thus, future research requires a larger number of patients to confirm our findings that the clinical outcome in patients with HRD-negative non-miliary HGOC may be similar to patients with HRD-positive HGOC. While initial efforts showed promising results [8,10], the future perspective should also focus on the introduction of biomarkers able to distinguish objectively between the miliary and non-miliary phenotype.

Despite some limitations our results indicate that in a predominantly PARPi naïve cohort, the type of tumor spread might be a better predictor of response to platinum sensitivity than HRD. It remains to be determined whether this also applies for sensitivity to PARPi.

## 5. Conclusions

Using a newly developed score for HRD assessment, PIGIS, we have demonstrated that tumors with miliary type tumor spread are HRD-negative and have a poorer prognosis than non-miliary spreading tumors. This indicates that in our predominantly PARPi naïve cohort, the type of tumor spread (miliary vs. non-miliary) might be a better predictor of the response to platinum-based therapy than HRD and that HRD is rather a surrogate marker for tumor spread. It remains to be demonstrated if this is also the case for PARPi based therapy.

## Figures and Tables

**Table 1:** Inclusion and exclusion criteria for the cohort.

**Inclusion criteria**	BRCA1/2 mutational status
	HGOC histology
**Exclusion criteria**	FIGO Stage Ia
	Variant of uncertain significance in BRCA1/2
	Unclear or to low tumor cell content in the sample
	Patients lost in follow-up
	Death unrelated to ovarian cancer diagnosis (2nd malignancy, accident, etc.)
	Complete documentation of treatment not available
	Peritoneal spread type not characterized

**Table 2:** Clinicopathologic parameters.

Variable	Patients (*n* = 122)	
	*n*	%
**ECOG Performance Status**		
0	86	70.5
1–2	33	27.2
≥3	3	2.3
**Histologic Subtype**		
HGS	109	89.3
Endometrioid	13	10.7
**Grading**		
Grade 2	42	34.4
Grade 3	80	65.6
**FIGO Classification**		
Ic	5	4.1
IIa–IIb	5	4.1
IIIa–IIIc	88	72.1
IVa	10	8.2
IVb	14	11.5

HGS = high-grade serous, ECOG = Eastern Cooperative Oncology Group, FIGO = International Federation of Gynaecology and Obstetrics.

**Table 3:** Patient treatment characteristics.

Variable	Patients *n* = 122	
	*n*	%
**Surgery**		
PDS	95	77.9
IDS	27	22.1
**Residual Disease**		
No residual disease	93	76.2
Residual < 1 cm	24	19.7
Any residual	5	4.1
**Adjuvant CTx**		
Carbo + Pacli	99	81.1
Carbo Mono	6	4.9
Carbo + PLD	3	2.5
Cis + Pacli ip	8	6.6
Carbo + Pacli + IO	6	4.9
**Maintenance**		
No therapy	56	45.9
Bev	48	39.3
PARPi	4	3.3
Bev + PARPi	8	6.6
Bev + IO	4	3.3
Bev + PARPi + IO	2	1.6
**Recurrence**		
No	38	31.1
Yes	84	68.9
Platinum Option *		
No (PFI < 6 Mo)	15	17.9
Yes (PFI ≥ 6 Mo)	69	82.1
**Death**		
No	73	59.8
Yes	49	40.2
**Mutational Status**		
WT	73	59.8
*BRCA1*	34	27.9
*BRCA2*	10	8.2
*RAD51C*	5	4.1
**Peritoneal Spread**		
Non-miliary	91	74.6
Miliary	31	25.4

PDS = primary debulking surgery, IDS = interval debulking surgery, WT = wild type, BRCA = breast cancer gene, *RAD51C* = radiation sensitive protein 51 paralog C gene, PFI = progression-free interval, Carbo = carboplatin, Pacli = paclitaxel, Bev = bevacizumab, * in recurrent disease (*n* = 84).

## Data Availability

Any requests for data by qualified scientific and medical researchers for legitimate research purposes are to subject to the Medical University of Innsbruck’s data sharing policy. All requests should be submitted in writing to Simon Schnaiter.
